# Progress in Medicine

**Published:** 1894-10-27

**Authors:** 


### Progress in Medicine.

### INFECTIVE DISEASES (*Continued*).

**Cholera.**—The Convention of Dresden[1] agreed that notification of the existence of a "foyer" of cholera should be given by an infected country to others though isolated cases should not be subjected to this rule. Soiled linen and a few other articles coming from an infected district must be disinfected, but, ordinary articles of merchandise are exempt. Persons actually sick of cholera in a vessel arriving at a port are to be landed and isolated, and the rest of those on board are to be landed and subjected to surveillance for not more than five days. The Congress at Rome[2] declared its adherence to the decisions of the Conference, with the condemnation of quarantine on land and of the restrictions on railway trains which they also embodied.

The Indian Government[3] is actively employed in regulating the great religious fairs, so that they may not become nurseries of cholera. The most perfect sanitary arrangements were made for the fair at Allahabad in February, where 3,000,000 pilgrims were collected, and the people seemed to thoroughly appreciate the supply of pure water and other preparations for their safety. Enormous platforms have been made at Hardwar[4] for the bathers, and similar measures are being elsewhere carried out.

The terrible mortality in the Mecca pilgrimage from cholera, amounting to 60,000 deaths last year, and the spread of cholera through the world in consequence, were brought before the attention of Europe by the paper of Mr. Ernest Hart last year, and the disgraceful state of Camaran,[5] the quarantine station for the arriving pilgrims, is the subject of stringent regulations by the Paris Convention. A subsequent discussion arose as to the possibility of forbidding the pilgrimage to pauper Moslemen. Though the Koran appears to excuse them from the pilgrimage, the Turkish delegate said it was unlawful for a Moslem

Government to forbid them the undertaking. The Moulvie Rafiuddin Ahmad[6] contributed to the *British Medical Journal* an able paper on the duty of the English and Turkish governments in the matter, and on the feeling excited among Moslems by the present state of things. The declaration of the Koran that the pilgrimage is incumbent on all who are able to find their way thereto is explained for many of their sects to imply the possession of means for their own support and that of their families while absent. Throughout the summer cholera has spread over Europe for the third year, actually in excess of the outbreak of the preceding year. It appeared in Russia, Austria, North-East Germany, and Belgium, as well as Portugal and Sweden,[7] but in England we have had very few even of suspicious cases. It is hard to say how far these attacks depend on the revival of old germs or of fresh infections. Even in India[8] the disease does not remain permanently in one place, but dies out and wanders here and there, reappearing after a period of quiescence when infection occurs under favourable conditions. Chantmesse has given a careful account of two mild, but extensive, epidemics in Constantinople and Lisbon.[9] The latter is of interest from the closeness of the resemblance of the clinical symptoms and of the characters of the comma bacillus present to those of true Asiatic cholera, while the mortality was almost nil. The bacillus seemed to agree exactly in its properties with the Finkler Prior one, and its relations to the Koch bacillus thus became of extreme importance. Besides the fact that healthy persons may have within them the Koch bacillus without clinical symptoms, unless special conditions favour its action, there are very varying types of Asiatic cholera, due to modifications of the bacillus which tend to be permanent for considerable periods. Next there is the contagious, but attenuated, type called cholera nostras, which is probably due to the Finckler Prior organism, as well as non-contagious choleriform diarrhœa with no bacilli at all. Chantmesse classes the Lisbon outbreak as an instance of cholera nostras.

**Scarlet Fever.**—J. Boehm[10] in the Tübingen laboratory has reviewed the discussion on the bacteriology of this disease, and denies the assertion of Babes that the streptococcus is the primary pathogenic factor in its etiology. Babes had been led to this view by the constant presence of the streptococcus in thirteen successive cases of glomerulo-nephritis; but Boehm points out that the kidney changes do not develop simultaneously with the invasion of streptococci. He does not find any organism uniformly present in relation to the scarlatinal process, and believes the streptococcus is merely a secondary infection. Washbourne[11] in his research, with the support of the British Medical Association funds, finds a streptococcus, which is apparently the one known as pyogenes, uniformly present in the pus and exudations. Nothing was discovered in the blood, and no diphtheria bacilli in the primary exudation on the tonsils, though they were present in all cases of membrane occurring as a sequela.

W. Gibson[12] writes on the possibility of disinfecting scarlatina patients in the second or third week by bathing in water to which disinfectants had been added. He thinks this may be done so completely that they may then mix with other people, though the desquamation is by no means complete, and refers to several cases which he had thus treated. An epidemic of 89 cases at Blackheath[13] has been examined and traced to a milk supply by Mr. Shirley Murphy, but how the milk became infected is not by any means clear. About 100 similar outbreaks due to milk infection have now been recorded. At Richmond Hill[14] yet another epidemic occurred of 55 cases. Here $\frac{17}{18}$ths of the cases were within the area of a milk supply, which served less than $\frac{1}{14}$th of the houses in Richmond. An investigation into the source of the supply was too late to be of any use in finding the original starting-point of the disease. A scarlet rash has been noted by Suckling[15] and others as occasionally appearing after enemata of warm water, and as due to the absorption of fœcal matter liquified by the injection. It is almost exactly like that of scarlatina, and may rarely be accompanied by a sore throat and slight fever. It lasts about twenty-four hours and may be distinctly urticarial.

**Actinomycosis.**—Wolf and Israel[16] have cultivated the microbe in alkaline bouillon, and obtained a growth consisting of rods, wavy filaments, and felt-like masses, but showing no club-shaped bodies. The latter appeared when the culture was injected into animals, and out of 22 inoculations 21 were successful. Netter[17] showed a patient cured by the administration of iodide of potash. She had developed a scrofulous pleurisy and a nodule over the ribs containing pus. Microscopic examination showed the presence of the special organism. He notes that 71 out of 185 oxen were cured in Chicago by the same drug, and six successful cases are reported from Holland. The drug must be given in full doses—80 grains daily at first, and may be reduced after a time. Illich[18] has collected in his work on Actinomycosis nearly all the recorded cases since Israel drew attention to it in 1885. He discusses in all 421 cases, and relies chiefly on incision and scraping. Where the viscera are affected iodide of potash treatment may be of value. On the whole he regards the disease as one which ultimately, if not readily, yields to careful treatment.

**Anthrax.**—A report[19] on the prevalence of this disease in London was recently made to the County Council. A hundred and eighteen cases are known to have occurred there since 1873. It has decreased in tanyards owing to the increased use of wet hides over dry ones. The importance of early recognition and treatment by excision is insisted upon, as at present it seems impracticable to disinfect all hides. H. Langley Browne[20] treated a case at West Bromwich by painting with iodine and a compress of corrosive sublimate of 1 in 1,000 strength. The slough separated from the rest of the patient's cheek, and a cure resulted. Diatroptoff succeeded in demonstrating the bacilli from the mud of a well where an outbreak of anthrax had occurred.[21] The water of the well had been suspected of infecting sheep on several occasions, and our author found in it bacilli which killed rabbits with symptoms of anthrax. From them fresh cultures were obtained.

A very elaborate report on anthrax is given by Dr. H. L. Burrell.[22] He concludes that complete excision of the pustule, where possible, is the best method of treatment. If this is impossible, injection of the most energetic antiseptic may be used, but glandular swelling about the pustule and apparent systemic poisoning do not counterindicate operative treatment. There may be cases without any pustules, or where these develop internally, as in an instance where intestinal obstruction was diagnosed. The pustule may be replaced by an erythematous or œdematous patch, a bubo, or phlegmon. Immunity may be conferred by inoculation or a cure effected by the injection of certain other microbes, such as the erysipelas streptococcus or the injection of carbolic acid or bichloride around and into the pustule. More than 100 cases have been thus cured. Dr. Abbe[23] gives another report upon a case which ended fatally after excision and packing with iodoform gauze. In the discussion Wyeth upheld incision with injection of bichloride.

Sestini[24] reported a successful case healed by injections of phenol and bichloride dressings. Müller[25] is against incision, as causing a wider systemic infection by the blood. He would fix the limb, which should be elevated, and apply mercurial ointment, with nutritious, stimulating diet. All the cases thus treated in Bramann's clinic since 1890 have recovered. Emmerich[26] discusses the various forms of serum treatment, and speaks highly of filtered serum from erysipelas in sheep. Sanarelli,[27] agreeing on the whole with Metchnikoff, denies that healthy lymph has a bactericidal power over the spores. He found that they grew in it luxuriantly at first, but soon died out, as they do in other media.

Gramatchikoff[28] appears to deny the efficacy of the extracts of the thymus or testicle in conferring immunity against anthrax, contrary to several earlier researches. Ilkewicz[29] has stained what he regards as the nuclei of anthrax spores. The protoplasm of the bacillus under his method is stained a dark grey, showing a granular structure. The spores vary in size, and may contain one or two nuclei. Gratia[30] points out that the segments of the bacillus colony are slightly enlarged at each end, and that a well-marked capsule envelops the entire rod or chain. This capsule is lost when the organism is grown *in vitro*, and its diagnostic value is great against other germs from the cadaver, and especially from the bacillus of malignant œdema.

[1] Practit., May, 1894. [2] Lancet, April 7th. [3] Lancet, April 7th. [4] B.M.J., May 19th. [5] B.M.J., Feb. 7th. [6] B.M.J., April 28th. [7] B.M.J., Aug. 28th. [8] B.M.J., July 28th. [9] Pract., July; Med. Week, June 22nd. [10] Int. Med. Mag., June. [11] B.M J., July 28th. [12] Practit., July. [13] B.M.J., May 11th. [14] Lancet, June 2nd. [15] B.M.J., June 2nd. [16] Med. Rec., May 5th. [17] Ther. Gaz., Jan. [18] Lancet, April 7th. [19] Lancet, July 28th. [20] B.M.J., July 14th. [61] Med. Rec., Dec. 2nd, 1893. [22] Annals Surgery, Dec., 1893. [23] Annals Surgery, July, 1894. [24] B.M.J., March, 1894. [25] B.M J., July 21st, 1894 [26] B.M.J., July 28th, 1894. [27] B.M.J., March 10th, 1894, [28] B.M.J., March 10th, 1894. [29] B.M.J., May 19th, 1894. [30] B.M.J., May 4th, 1894.

